# Childhood caries is associated with poor health and a faster pace of aging by midlife

**DOI:** 10.1111/jphd.12591

**Published:** 2023-11-02

**Authors:** Begoña Ruiz, Jonathan M. Broadbent, W. Murray Thomson, Sandhya Ramrakha, Terrie E. Moffitt, Avshalom Caspi, Richie Poulton

**Affiliations:** ^1^ Sir John Walsh Research Institute, Department of Oral Sciences, Faculty of Dentistry University of Otago Dunedin New Zealand; ^2^ Dunedin Multidisciplinary Health and Development Research Unit, Department of Psychology, Division of Sciences University of Otago Dunedin New Zealand; ^3^ Department of Psychology and Neuroscience Duke University Durham North Carolina USA; ^4^ Department of Psychiatry and Behavioral Sciences Duke University Medical Center Durham North Carolina USA; ^5^ Center for Genomic and Computational Biology Duke University Durham North Carolina USA; ^6^ Social, Genetic, and Developmental Psychiatry Centre, Institute of Psychiatry Kings College London London USA

**Keywords:** aging, birth cohort, cohort studies, dental caries, epidemiology, health, health status, public health, public health dentistry

## Abstract

**Objectives:**

Childhood caries is associated with poorer self‐rated general health in adulthood, but it remains unclear whether that holds for physical health and aging. The aim of this study was to identify whether age‐5 caries is associated with (a) biomarkers for poor physical health, and (b) the pace of aging (PoA) by age 45 years.

**Methods:**

Participants are members of the Dunedin Multidisciplinary Health and Development Study birth cohort. At age 45, 94.1% (*n* = 938) of those still alive took part. Data on age‐5 caries experience and age‐45 health biomarkers were collected. The PoA captures age‐related decline across the cardiovascular, metabolic, renal, immune, dental and pulmonary systems from age 26 to 45 years. We used (a) generalized estimating equations to examine associations between age‐5 caries and poor physical health by age 45 years, and (b) ordinary least squares regression to examine whether age‐5 caries was associated with the PoA. Analyses adjusted for sex, perinatal health, childhood SES and childhood IQ.

**Results:**

High caries experience at age‐5 was associated with higher risk for some metabolic abnormalities, including BMI ≥30, high waist circumference, and high serum leptin. Those with high caries experience at age‐5 were aging at a faster rate by age 45 years than those who had been caries‐free.

**Conclusions:**

Oral health is essential for wellbeing. Poor oral health can be an early signal of a trajectory towards poor health in adulthood. Management for both conditions should be better‐integrated; and integrated population‐level prevention strategies should be foundational to any health system.

## INTRODUCTION

Non‐communicable diseases (NCDs) are highly prevalent; their global burden, together with that of mental health conditions, constitutes a major societal public health problem. NCDs—including cardiovascular diseases, cancer, diabetes, and chronic respiratory diseases—are the leading cause of premature death (death between ages 30 and 70 years), and account for seven out of ten deaths globally.^1^ A similar pattern holds in New Zealand (NZ) and 89.0% of deaths reported in 2016 arose from NCDs. Additionally, the global burden of disease and disability is increasing as the global population gets older. Individuals aging faster are less fit, become less physically able, show greater cognitive decline and brain aging, and even look older than those aging more slowly.^2^

The World Health Organization's recent Global Oral Health Strategy recognized oral diseases as NCDs and highlighted their magnitude (more than 3.5 billion cases of oral diseases globally in 2017) and impact on individuals and society. It stated that the global prevalence of dental caries, periodontal disease and tooth loss combined is higher than that of any other NCD.^3^ It also claimed that there is evidence for associations between oral diseases (particularly periodontal disease) and a range of other NCDs, such as diabetes and cardiovascular disease, notwithstanding inconsistencies in the quality of published, available data on those. Differences in study designs, participant populations, selection criteria, measurement, and reporting of outcome measures have led to heterogeneity in findings and conclusions. Recent findings from two oral health birth cohort studies found no associations between periodontitis and markers for cardiometabolic risk^4^, ^5^ or dysglycaemia, at least into early adulthood.^6^

There is a need for more high‐quality evidence to determine whether oral health has a detectable impact on general health. Claiming that association implies causation would be incorrect and misleading,^7^ considering that oral conditions share common risk factors—including high sugars intake (and lack of breastfeeding), smoking, harmful alcohol use—and co‐occur with other NCDs,^8^ and that there are commonalities in the social determinants of both oral and general ill‐health.^9^

The lifecourse approach has been emphasized as an important framework giving context to the most recent WHO definition of oral health, in support of the dynamic and cumulative nature of oral conditions, and as a key principle of the ‘Global Action Plan’ for preventing NCDs. This sociological framework transcends cross‐sectional associations, highlighting that the exposure to risk factors for NCDs begins in childhood. This is exemplified by observations that socioeconomic disadvantage^10^ and low IQ^11^, ^12^ are associated with poor mental and physical outcomes in later life.

We recently reported that greater caries experience in childhood was associated with poorer oral health and self‐reported general health by midlife.^13^, ^14^ Here, we extend the reach of such research by examining associations between childhood oral health and general health in midlife. Using data from NZ's Dunedin Multidisciplinary Health and Development Study, we investigated whether caries experience at age five years is associated with biomarkers for poor physical health and higher pace of aging by age 45.

## METHODS

### Study design and population

Participants were members of the Dunedin Multidisciplinary Health and Development Study (hereafter, Dunedin Study), a longitudinal investigation of a population‐representative birth cohort of 1037 individuals (91% of eligible births; 52% boys) born between April 1972 and March 1973 in Dunedin, NZ. The cohort represented the full range of socioeconomic status (SES) of NZ's South Island, and is primarily NZ European (93%), matching South Island demographics. Participants have been assessed on physical and mental health domains, social circumstances, development, and wellbeing. General assessments took place at birth, then ages 3, 5, 7, 9, 11, 13, 15, 18, 21, 26, 32 and 38 years and, most recently (completed April 2019) at age 45 years, when 94.1% (*n* = 938) of the 997 surviving study members took part. Written informed consent was obtained from parents (from ages 3 to 13 years), and from all participants in the remaining assessments ages (15–45 years). Each assessment was approved by the appropriate ethics committee, most recently the NZ Health and Disability Ethics Committee (17/STH/25/AM05).

### Childhood caries at age five years

The present study used data on deciduous dentition caries experience. For some analyses, study members were classified as caries‐free (dmft = 0), moderate caries experience (dmft = 1–4) or high caries experience (dmft ≥5). For details on previous dental examinations, see Data S1 (Explanatory variables and covariates).

### Physical health at age 45 years

Physical examinations were conducted at the Dunedin Study research unit, with measurement of biomarkers taken in counterbalanced order with the exception of blood, which was drawn at the same time of day for all. In the present study, physical health was represented by 17 biomarkers, including clinical indicators of metabolic abnormalities (body mass index (BMI), waist‐hip ratio, waist circumference, glycated hemoglobin (HbA1c), serum leptin levels, mean arterial pressure, total cholesterol, triglyceride levels, high‐density lipoprotein levels, lipoprotein (a) levels, serum apolipoprotein B100/serum apolipoprotein A1 ratio), cardiorespiratory fitness, pulmonary function, kidney function (estimated glomerular filtration rate, blood urea nitrogen) and systemic inflammation (C‐reactive protein level, white blood cell count). For each biomarker, clinical cut‐offs based on international guidelines were used to define poor physical health. For biomarkers with no specific recommended cut points, sex‐specific quartiles were formed to identify those at risk.

To calculate the pace of aging (PoA), we used biomarker data from the age 26, 32, 38 and 45 assessments. The PoA is an indicator of the cumulative, progressive and gradual deterioration across organ systems that underlies biological aging. Descriptions for each biomarker, their clinical cut‐offs and the PoA variable are provided in the Data S1, Table S1.

### Covariates

Perinatal complications were recorded shortly after birth, with study members classified as 0 with none, or as 1+ where there were ≥1 perinatal complications. Childhood SES was estimated as the average of the highest level of either parent using the Elley‐Irving scale of occupational status, which had been assessed at the study member's birth and at ages 3, 5, 7, 9, 11, 13, and 15 years. Individuals were allocated to high, medium, and low SES categories. Childhood IQ was assessed using the Wechsler Intelligence Scale for Children–Revised (WISC–R), administered at ages 7, 9, and 11 years. The IQ variable used for the current analyses was the averaged measure of IQs determined at these three ages, standardized to population norms with a mean of 100 and a standard deviation of 15. For detailed description of covariates, see Data S1.

### Statistical analysis

Generalized estimating equations (GEEs) were used to estimate associations between primary dentition caries experience at age five and poor physical health outcomes measured at age 45 years. Poisson regression using GEEs (with robust variance estimation and independent working correlation) estimated incidence rate ratios for each biomarker according to clinical thresholds. All analyses adjusted for sex, perinatal health, childhood SES and childhood IQ and were guided by a directed acyclic graph (Figure S1). To ensure the findings' robustness, analyses were repeated using quantile regression to examine whether the association with age‐5 dental caries differed along the distribution of each of the health biomarkers. Ordinary least squares linear regression was used to examine whether age‐5 caries experience was associated with the PoA at age 45 years. Analyses used Stata/SE 17.0 (StataCorp LLC, College Station, Texas, USA). Data reporting followed STROBE guidelines.

## RESULTS

Dental data at age five were available for 922 participants (50.3% males); the proportion of caries‐affected children was 59.1%, and the mean dmfs was 3.7 (s.d. = 5.9). Data on physical health biomarkers at age 45 were available for BMI (*n* = 920), waist‐hip ratio (*n* = 904), waist circumference (*n* = 905), HbA1c (*n* = 876), leptin (*n* = 875), MAP (*n* = 906), VO_2_max (*n* = 849), FEV1/FVC (*n* = 888), total cholesterol (*n* = 879), triglycerides (*n* = 879), HDL (*n* = 877), lipoprotein(a) (*n* = 878), ApoB/apoA1 (*n* = 874), eGFR (*n* = 875), BUN (*n* = 879), hsCRP (*n* = 841), white blood cell count (*n* = 876) and PoA (*n* = 955).

Consistent gradients were observed in mean values for BMI, waist circumference, leptin levels, c‐reactive protein, white blood cell count and the pace of aging by dental caries experience at age five (Table 1). These were greater among those with high caries experience and lower among those who were caries‐free as children (*p* < 0.05). An expected opposite gradient was observed for respiratory health, with higher cardiorespiratory fitness (VO_2_Max) indicating better fitness.

**TABLE 1:** Mean values for biomarkers at age 45 year by age‐5 caries experience categories.

	Dmft 0 mean (SD)	Dmft 1–4 mean (SD)	Dmft 5+ mean (SD)	Effect size^b^
Physical health biomarker				
Metabolic abnormalities				
BMI (kg/m^2^)	27.6 (5.1)	28.7 (6.3)	29.6 (5.4)^a^	0.4 (Moderate)
Waist‐hip ratio	0.9 (0.1)	0.9 (0.1)	0.9 (0.1)	—
Waist circumference (cm)	90.0 (13.7)	93.2 (15.3)	95.1 (14.0)^a^	0.4 (Moderate)
HbA1c (%)	5.7 (0.4)	5.7 (0.7)	5.7 (0.4)	—
Leptin (μg/L)	20.2 (22.2)	22.8 (24.8)	26.9 (27.4)^a^	0.3 (Moderate)
MAP (mmHg)	93.9 (11.2)	94.3 (11.9)	94.0 (11.4)	—
Total cholesterol (mmol/L)	5.2 (1.0)	5.1 (0.9)	5.3 (1.0)^a^	0.1 (Small)
Triglycerides (mmol/L)	2.1 (1.4)	2.2 (1.6)	2.1 (1.3)	—
HDL cholesterol (mmol/L)	1.6 (0.5)	1.5 (0.4)	1.5 (0.4)	0.2 (Moderate)
Lipoprotein (a)	402.8 (425.1)	399.8 (419.0)	518.8 (597.7)	0.3 (Moderate)
ApoB/ApoA1	0.6 (0.2)	0.6 (0.2)	0.6 (0.2)	—
Respiratory health				
VO_2_Max	27.4 (7.2)	26.8 (7.5)	25.6 (7.5)	0.3 (Moderate)
FEV_1_/FVC	78.4 (6.7)	78.3 (6.6)	79.0 (5.6)	0.1 (Small)
Kidney function				
eGFR (ml/min/1.73m^2^)	86.0 (6.3)	87.2 (5.5)	86.9 (5.7)	0.1 (Small)
BUN (mmol/L)	5.3 (1.2)	5.3 (1.5)	5.4 (1.1)	0.1 (Small)
Systemic inflammation				
hsCRP (mg/L)	1.8 (2.0)	1.8 (2.0)	2.3 (2.1)^a^	0.3 (Moderate)
White blood cell count (×10^9^cells/L)	7.6 (1.8)	7.7 (1.9)	8.0 (1.9)^a^	0.2 (Moderate)

Abbreviations: ApoB/ApoA1, serum apolipoprotein B100/serum apolipoprotein A1 ratio; BMI, body mass index; BUN, blood urea nitrogen; eGFR, estimated glomerular filtration rate; FEV_1_, forced expiratory volume in one second; FVC, forced vital capacity; HbA1c, glycated hemoglobin; HDL, high‐density lipoprotein levels; hsCRP, serum C‐reactive protein; MAP, mean arterial pressure; SD, standard deviation; VO_2_max, predicted maximum oxygen uptake adjusted for body weight in milliliters per minute per kilogram.

^a^ Kruskal‐Wallis test, *p* < 0.05.

^b^ Effect size = [(dmft5+) − (dmft0)]/sd(dmft0); <0.2 = small; 0.2–0.7 = moderate; >0.7 = large.

**FIGURE 1:**
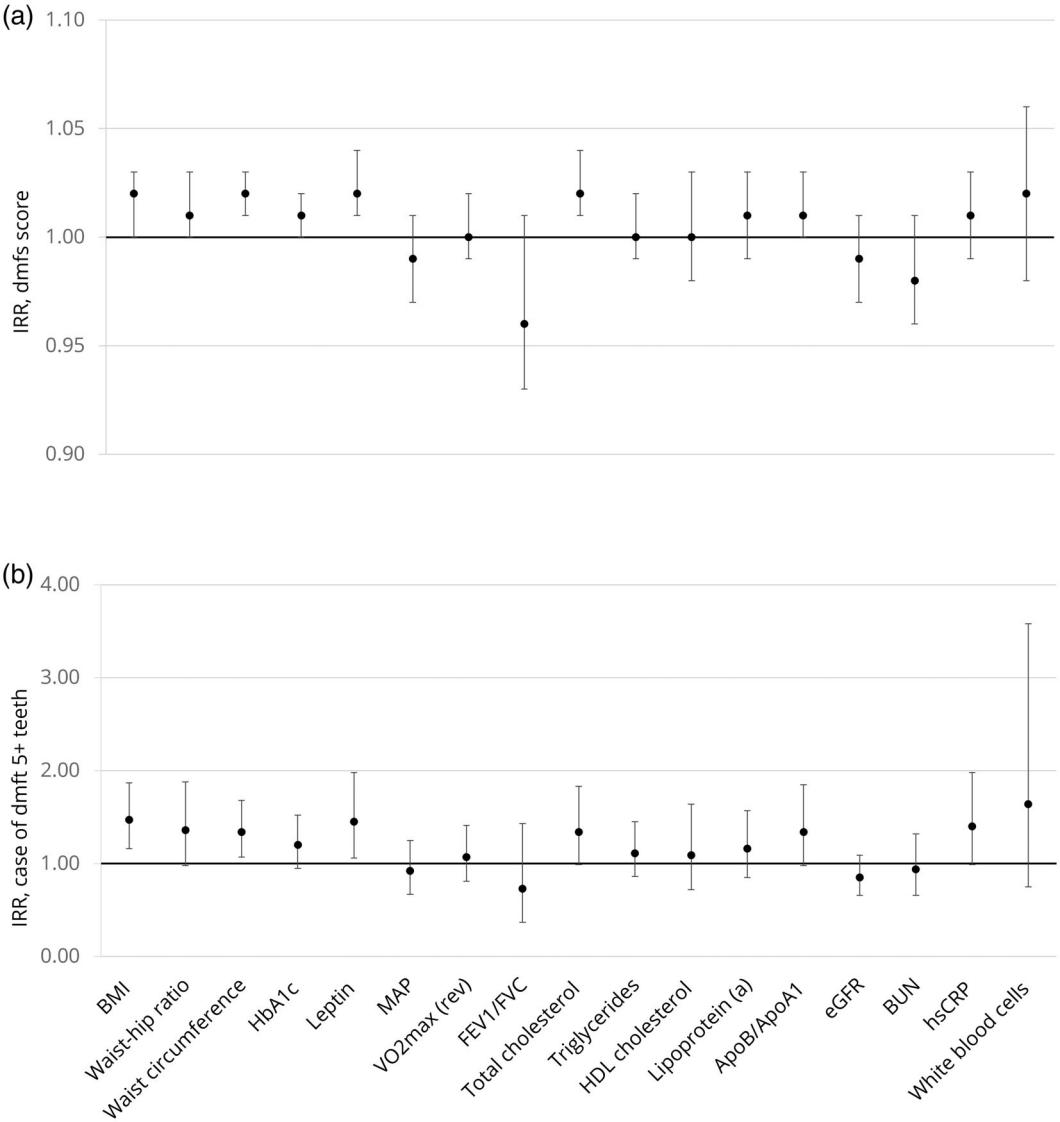
Associations between age‐5y caries experience and general health biomarkers at age 45y among Dunedin study participants. (a), Poisson regression model for biomarker thresholds by dmfs score at age five years. (b), Poisson regression model for biomarker thresholds by high caries experience (5+ caries‐affected teeth) at age five years, comparison group = caries‐free.

Higher caries experience at age five years (5+ caries‐affected teeth) was associated with metabolic abnormalities by age 45 years, including BMI ≥30, high waist circumference (≥88 cm for women, ≥102 cm for men), and being in the highest quartile for serum leptin (Figure 1), even after accounting for sex, perinatal health, childhood socioeconomic status and childhood IQ. Specific associations between age‐5 caries experience and each health biomarker are presented in Tables S2–S5 and Figure S2. Consistent findings were observed with different thresholds for caries experience at age five years and when associations were examined by using quantile regression models (Tables S6–S9). Adjustment for adult socioeconomic status did not result in any meaningful differences for the observed associations (data not shown).

We observed a gradient in the PoA to age 45 whereby participants who had higher caries experience at the age of five years were aging at a faster rate than those who were caries‐free. For participants who as children were caries‐free, mean PoA was 0.95 (95% CI, 0.92, 0.98); for those with moderate caries (dmft 1–4), the mean PoA was 1.01 (95% CI, 0.98, 1.05); and, for those who had high caries experience (dmft 5+), the mean PoA was 1.07 (95% CI, 1.03, 1.12). The adjusted analyses showed that participants who had high caries experience at age five years were aging 0.1 years of physiological change per chronological year faster by age 45 than those who were caries‐free (Table 2). To investigate whether childhood dental caries was associated with the PoA regardless of the presence of adult dental caries in the PoA measure, we examined associations between dental caries at age five years and the PoA at age 38 years. The latter measure did not include carious surfaces as part of the biomarkers. Consistent findings were observed whereby 5+ caries‐affected teeth or ‘any caries’ were associated with PoA to age 38 years, but dmfs/dmft scores and PoA to age 38 years were no longer associated (Table S10). Adjustment for adult SES did not mitigate the observed associations.

**TABLE 2:** Associations between caries experience at age‐5‐years and the pace of aging (PoA) among Dunedin study participants at 45 years of age.

	Unadjusted (*n* = 860)			Adjusted for sex, childhood SES and childhood IQ and perinatal health (*n* = 857)		
	*β*	*β* 95% CI	*p*	*β*	*β* 95% CI	*p*
Caries experience at age 5 years						
dmfs	0.006	0.003, 0.009	<0.001	0.004	0.001, 0.008	0.004
dmft	0.014	0.008, 0.020	<0.001	0.011	0.005, 0.016	<0.001
Caries‐free	Ref.					
dmft 1–4	0.064	0.022, 0.107	0.003	0.050	0.009, 0.092	0.016
dmft 5+	0.123	0.072, 0.175	<0.001	0.102	0.052, 0.152	<0.001
Caries‐free	Ref.					
Any caries	0.085	0.046, 0.123	<0.001	0.068	0.030, 0.106	<0.001

Abbreviations: *β*, ordinary least squares regression beta coefficients; CI, confidence interval; Ref., reference category (comparison group = dmft = 0).

## DISCUSSION

This lifecourse study investigated whether childhood dental caries was associated with biomarkers of poor physical health by middle‐age while considering important early‐life determinants of health, including perinatal health, childhood social background, and childhood cognitive ability, finding that those with caries at age five were more likely to have metabolic abnormalities by midlife. Moreover, childhood caries at age five years was associated with a faster rate of biological aging across multiple systems. This study supports three main findings: (1) poor oral health and overweight/obesity share similar early‐life and subsequent risk factors; (2) there is continuity in experience of poor oral health in childhood and poor general health (such as abnormal fat accumulation) by midlife; and (3) early childhood caries is associated with an accelerated pace of biological aging in later life.

In this early‐life model, greater caries experience in childhood was associated with obesity and higher leptin levels by age 45 years. To date, only a few studies have reported longitudinal associations between dental caries (as predictor) and measures of obesity (as the outcome). Our findings extend those of a recent longitudinal study of oral health and physical function among older adults (aged 70+ years and followed for about 8 years) that found oral health conditions, including self‐reported dry mouth and periodontal disease, to indicate risk for decline in muscle strength and performance in later life.^15^ Another found no association between parental report of children's dental problems and BMI after a 4‐year follow‐up in a large representative sample of 4149 children aged 6–7 in the Longitudinal study of Australian Children.^16^ Two other studies have shown inverse associations between caries and anthropometric measures. In England, Hall‐Scullin et al. showed that caries experience—DMFT>0 at ages 7–9 years—was not associated with overweight or obesity by ages 12–16.^17^ However, there was some evidence of differential loss to follow‐up. Likewise, an inverse association between caries at baseline and subsequent changes in BMI over 6 years was observed among 8‐10‐year‐old Danish children, but only in those whose mothers were well educated,^18^ in whom there was a smaller BMI increment for children with high caries experience. Conversely, for children with less‐educated mothers, higher caries experience was associated with a larger body weight increment (albeit not statistically significant, in a small group). That the cohort was in the midst of the transition from primary dentition to the permanent one may also have affected the chance of uncovering an association.

Our findings are consistent with those of a representative sample of Hong Kong 12‐year‐olds examined and followed over 6 years, which reported association between DMFT at age 15 and central obesity at the age of 18 years.^19^ More recently, severe dental caries experience was associated with both thinness and overweight in a sample of Chinese pre‐schoolers.^20^

Some have proposed that poor dental health may adversely affect masticatory function, favoring the choice of low‐fiber, energy‐dense foods, leading to weight gain.^21^ Although this interpretation seems plausible, we believe that the positive associations between childhood dental caries and measures of obesity observed some five decades later in the present study were to be expected and are better explained by both conditions sharing important common risk factors. Diet—and more specifically, the excessive consumption of sugars—is one of them, but, most importantly, they share the same underlying psychosocial, economic, environmental and political determinants.^22^, ^23^

We observed childhood caries experience to be associated with higher leptin levels by age 45. Leptin, an adipocyte‐secreted hormone, regulates appetite and promotes energy expenditure. Hyperleptinemia is positively associated with central obesity and insulin resistance.^24^ Obese individuals are likely to develop leptin resistance, resulting in greater food intake perpetuating the progression of obesity. Also, leptin and insulin regulate each other in blood‐glucose homeostasis, whereby restricted leptin signaling can induce hyperglycaemia and hyperinsulinemia, ultimately leading to diabetes mellitus.^25^ Sugar might be the common factor driving dental caries and metabolic abnormalities in this population.

Obesity is a complex condition that produces a low‐grade inflammatory state with higher metabolite levels. Adipose inflammation is associated with systemic inflammation and lower insulin sensitivity. Overweight or obese individuals have higher levels of inflammatory cytokines such as hs‐CRP, serum amyloid A, tumor necrosis factor‐alpha, IL‐6, IL‐1β, and IL‐8.^26^ We did not observe statistically significant associations between dental caries experience at age five years and markers of systemic inflammation (hsCRP and white blood cell count). It may be that this (and those with other health variables) emerges later in life.

Dental caries and obesity are both highly prevalent conditions that are socially patterned. They have complex etiology involving a substantial behavioral component. Food environments are major drivers of unhealthy diets and energy overconsumption.^27^ Obesity is the result of people responding normally to the obesogenic environments in which they find themselves.^23^ In the same way, poor oral health and oral health inequalities exist because of socially‐determined differences in opportunity, behaviors, beliefs, and exposure to the various factors that are structurally determining oral health.^28^ Childhood socioeconomic background predicts adult morbidity and mortality. In this cohort, childhood SES was associated with poorer health in midlife even after controlling for adult SES. The social gradient in health emerged in childhood and was not mitigated by upward mobility.^10^ For this reason, we presented a model with childhood covariates only, in a way to portray an ‘early‐life model’. No meaningful differences were observed for any of the associations found after considering the effects of adult SES.

Another NZ longitudinal study has reported obesity to be associated with a complex mix of childhood family background, biological endowment and individual factors beginning in pregnancy and early childhood.^29^ We acknowledge that genetic endowment plays a role in food‐liking and that it might influence choices, behavior and intake. However, research in this area is limited and associations between variation in sweet or fatty‐taste perception and measures of intake or obesity have yet to be elucidated.^30^

Those who had experienced dental caries by age five aged more rapidly than those who were caries‐free then. This might be explained by considering that dental caries in childhood—as a chronic, cumulative condition—is associated with experience of disease in adulthood and that the PoA at age 45 included caries‐affected tooth surfaces as part of the biomarkers assessed at ages 26, 32, 38 and 45 years. However, to determine whether poor oral health was associated with the PoA measure regardless of the presence of adult dental caries as part of the aging measure, we examined whether there was an association between age‐5 caries and the PoA variable by age 38 years (which had not included dental caries as part of the 18 biomarkers that constituted the indicator). This showed that childhood caries experience was positively associated with the PoA even when caries was not included in the aging indicators.

Previous findings from the same cohort showed an accelerated pace of aging to be associated with greater risk of showing signs of sensory motor difficulties, diminished sensory abilities, and poorer cognitive functioning.^31^ A faster pace of aging serves as a foundation for developing future illnesses. Consistent with this, the burden of NCDs is rising as the world population ages. In 2015, NCDs accounted for 18 of the leading 20 causes of age‐standardized years lived with disability globally.^32^ Developing countries are transitioning to a disease burden characterized by the ailments of aging societies, whereby cancers, ischaemic heart disease, cirrhosis, Alzheimer's disease, and injuries are responsible for the majority of years lived with disability.^33^ Greater life expectancy and NCDs are creating new challenges for health systems, and early detection and control of metabolic risk factors seems a priority for policy development.

Limitations of this study include that it involves a sample of primarily NZ European ethnicity and so findings can be generalized only to similar populations. Second, the Dunedin Study cohort has been followed so far only to 45 years, when most Study members (60%) self‐reported no physical health conditions,^13^ and so further investigation as they age is needed to elucidate whether new associations emerge. We acknowledge the paramount role of sugar consumption in the development of caries and NCDs. In the Dunedin Study, sugar consumption data collection has been limited and relates only to nocturnal drinks/snacks (at age 5); however, we did find this to be associated with higher caries experience cross‐sectionally. Nonetheless, this one‐timepoint sugar‐related variable was not included in our modeling strategy because it provided incomplete information. The main study strengths are: using unique prospective data from a population‐based cohort followed from birth to the fifth decade of life with high follow‐up rates (94%); caries experience recorded at both surface‐ and tooth‐level; using a broad battery of health measures commonly used to predict frailty, morbidity and mortality^31^; and using a dynamic indicator of progressive deterioration in health, the pace of aging variable.

In conclusion, childhood dental caries is associated with poorer general health and a faster rate of aging by midlife. These lifecourse findings support the assertions that: oral health is essential for wellbeing; poor oral health can be an early signal of a trajectory towards poor health in adulthood; management for both conditions should be better‐integrated; and integrated population‐level prevention strategies should be foundational to any health system.

## FUNDING INFORMATION

The New Zealand Health Research Council, the Ministry of Business, Innovation and Employment, the US National Institute of Aging and the UK Medical Research Council.

## CONFLICT OF INTEREST STATEMENT

The authors declare no conflict of interest.

## Supporting information


**Data S1.** Supporting Information.

## Data Availability

The Dunedin Study data are available on request to the Study Director by qualified scientists. Requests require a concept paper describing the purpose of data access, ethical approval at the applicant's institution, and provision for secure data access. We offer secure access on the Duke University, Otago University, and King's College London campuses. All data analysis scripts and results files are available for review.
