# Supplementary material for: Childhood caries is associated with poor health and a faster pace of aging by midlife
Source: J Public Health Dent. 2023 Nov 2;83(4):381–8. doi: 10.1111/jphd.12591 (PMC10919959; doi:10.1111/jphd.12591)
Supplement: Supplementary file 1 — Data S1. Supporting Information. [file JPHD-83-381-s001.docx]

**Supporting information**

**Childhood caries is associated with poor health and a faster pace of ageing by midlife**

**Study design and population**

Participants were members of the Dunedin Multidisciplinary Health and Development Study (hereafter, Dunedin Study), a longitudinal investigation of a population-representative birth cohort of 1037 individuals (91% of eligible births; 52% boys) born between April 1972 and March 1973 in Dunedin, NZ. The cohort represented the full range of socioeconomic status (SES) of NZ’s South Island, and is primarily NZ European (93%), matching South Island demographics (1). Participants have been assessed on physical and mental health domains, social circumstances, development, and wellbeing (2). General assessments took place at birth, then ages 3, 5, 7, 9, 11, 13, 15, 18, 21, 26, 32 and 38 years and, most recently (completed April 2019) at age 45 years, when 94.1% (n=938) of the 997 surviving study members took part. Written informed consent was obtained from parents (from ages 3-13 years), and from all participants in the remaining assessments ages (15-45 years). Each assessment was approved by the appropriate ethics committee (3), most recently the NZ Health and Disability Ethics Committee (17/STH/25/AM05).

**Explanatory variables and covariates**

Oral examinations were conducted at 5, 9, 15, 18, 26, 32, 38, and 45 years of age, with clinical data on dental caries, periodontal disease, oral hygiene, enamel defects and other oral health-related components collected accordingly. The present study used age-5 deciduous dentition caries experience data, with examinations conducted by dentists using WHO methods (4). Teeth were examined for dental caries and restorations, with four surfaces considered for canines and incisors, and five surfaces for premolars and molars. Caries experience was summarized using the dmf index at both surface and tooth levels (5). For some analyses, study members were classified as caries-free (dmft=0), moderate caries experience (dmft=1-4) or high caries experience (dmft≥5). This is consistent with the commonly-used service definition for high dental caries experience in NZ and with previous reporting (6). Details on previous dental examinations are described elsewhere (7–9). Sex, perinatal complications, childhood socio-economic status (SES), and childhood IQ were included as covariates based on previous research (10,11).

The term “sex” refers to the biological and physiological characteristics that define men and women. That is, “male” and “female” are sex categories. Many health conditions will manifest sex differences vary in their occurrence. Sex is considered an important determinant of health because it can affect disease risk, progression and outcomes through genetic and physiological pathways (WHO 2023). The term “gender” encompasses the roles, behaviours, activities and attributes that a given society considers appropriate for men and women (and boys and girls). Gender is a social construct and, in many contexts, it is a strong social determinant of health.

Perinatal health was used as a covariate because extensive research has shown that adverse conditions during *in-utero* growth (early development) have an impact on future chronic disease experience and early mortality. Thus, we included the measure of ‘perinatal complications’—assessed at birth—to account for the fact that some participants may have had ‘perinatal programming’ of a predisposition to poorer/worse health in midlife. In the Dunedin study, as previously described by (12) each child was examined shortly after birth, and perinatal information was taken from the hospital records. The obstetric complications assessed in this study, including prenatal, intrapartum, and neonatal complications, were maternal diabetes, glycosuria, epilepsy, hypertension, eclampsia, antepartum hemorrhage, accidental hemorrhage, placenta previa, having had a previous small baby, gestational age <37 weeks or >41 weeks, birth weight <2.5 kg, small size for gestational age, major or minor neurologic signs of the neonatal period (eg, jitteriness, tenseness, limpness, hypotonicity), Rh incompatibility, ABO incompatibility, non-hemolytic hyperbilirubinemia, hypoxia at birth (idiopathic respiratory distress syndrome or apnea), and low Apgar score at birth. The infant was defined as having a low Apgar score if 1 of the following conditions applied: At 5 minutes of life, the infant’s heart rate was 100 beats per minute, respiration was irregular or absent, and the infant was centrally cyanotic; the infant took >10 minutes to establish normal respiration; or the infant’s asphyxia at birth warranted resuscitation. The sum of maternal complications and neonate complications was significantly and positively correlated (r = 0.156, P< 0.001). Based on evidence that the effects of adverse conditions are cumulative, each condition was weighted equally and summed to yield an obstetric complications index. Study members were classified as 0 with none, or as 1+ where there were ≥1 perinatal complications.

Childhood socioeconomic background (SES) reflects the socioeconomic conditions experienced by the participants while they were growing up. This measure was used as a covariate because the social, economic and environmental conditions in which individuals and populations grow, live and work throughout the lifecourse greatly influence health (13,14). The wider determinants of health have been described as the ‘causes of the causes’. Social gradients exist not only for oral conditions (15–17) but in mortality and in most common chronic diseases, and the mechanisms to explain these gradients involve material, behavioural, environmental and psychosocial characteristics (15,18). Childhood socioeconomic status (SES) was estimated as the average of the highest level of either parent using the Elley-Irving scale of occupational socio-economic status (19) which places occupations into one of six categories ranging from 1=professional to 6=unskilled laborer. SES was assessed repeatedly at the study member’s birth and at ages 3, 5, 7, 9, 11, 13, and 15 years. Individual scores were trichotomised into high, medium and low categories.

Childhood IQ was used as a covariate because childhood cognitive ability is associated with health and longevity, health behaviours, ageing (20) and mortality (21,22). Childhood cognitive function is also a key determinant of oral health and dental service-use by midlife (23). Childhood IQ was assessed using the Wechsler Intelligence Scale for Children–Revised (WISC–R) (24) which was administered to the participants at ages 7, 9, and 11 years. The tests were individually administered at each assessment according to standard protocol. The IQ variable used for these analyses was the averaged measure of IQs determined at these three ages, standardized to population norms with a mean of 100 and a standard deviation of 15 (25).

**Biomarker description and clinical cut-offs**

As previously reported, measurement of biomarkers at age 45 years were taken in counterbalanced order across Study members with the exception of blood, which was drawn at the same time of day for all Study members (between 4:15 p.m. and 4:45 p.m) (16). Details on biomarker measurements and clinical cut-offs for the present analyses are provided in Table S1.

The pace of ageing (PoA) is an indicator of the cumulative, progressive, gradual deterioration across organ systems that underlies biological ageing. To calculate PoA we used biomarker data from the age 26-, 32-, 38- and 45 assessments (26,27). Women who were pregnant at the time of a given assessment were excluded from that wave of data collection.

**Table S1.** Description of biomarkers and clinical cut-offs for poor physical health among Study members of the Dunedin Multidisciplinary Health and Development Study at age 45 years.

| **Biomarker** | **Description** | **Reference** |
| --- | --- | --- |
| Body mass Index (BMI) | Height was measured to the nearest millimeter using a portable stadiometer (Harpenden; Holtain, Ltd.). Weight was measured to the nearest 0.1 kg using calibrated scales. Individuals were weighed in light clothing. BMI was calculated (kg/m^2^). High BMI (obesity) was defined as ≥30 kg/m^2^. | (28) |
| Waist-hip ratio | Waist girth was the perimeter at the level of the noticeable waist narrowing located between the costal border and the iliac crest. Hip girth was taken as the perimeter at the level of the greatest protuberance and at about the symphysion pubic level anteriorly. Measurements (in centimeters) were repeated and the average used to calculate waist-hip ratio. Sex-specific quartiles were formed based on waist-hip ratio at age 45 years. Study members in the highest quartile were considered to have High waist-hip ratio. | (29) |
| Haemoglobin A1c | Glycated haemoglobin concentration was measured in serum collected at age 45 years (expressed as a percentage of total haemoglobin) by ion exchange high performance liquid chromatography (Variant II: BioRad, Hercultes, Calif.), a method certified by the US National Glycohemoglobin Standardization Program (<http://www>.ngsp.org/). High glycated haemoglobin was defined as ≥5.7%, the cutoff for prediabetes (as previously reported in Israel et al. 2014 (20). | (30) |
| Leptin | Serum leptin (μg/L) was measured using the Quantikine ELISA Human Leptin Immunoassay (Cat# SLP00, R&D Systems Inc, Minneapolis, MN) (Age 45 years) according to the manufacturer’s instructions. Sex-specific quartiles were formed based on serum leptin at age 45 years. Study members in the highest quartile were considered to have High leptin levels. | (31) |
| Blood pressure | Blood pressure at age 45 years was measured in a quiet room with the study member in a seated position, by trained assesors. An automatic BpTRU Vital Signs Monitor BPM 200 (BpTRU Medical Devices, Canada). Mean arterial pressure (MAP) was calculated using the formula Diastolic Pressure+1/3(Systolic Pressure – Diastolic pressure). High blood pressure was defined as MAP >100mmHg. | (32) |
| Cardiorespiratory fitness (VO_2_Max) | Cardiorespiratory fitness was assessed by measuring heart rate in response to a submaximal exercise test on a friction-braked cycle ergometer. Dependent on the extent to which heart rate increased during a 2-min 50W warm-up, the workload was adjusted to elicit a steady heart-rate in the range 130–170 beats per minute. After a further 6-min constant power output stage, the maximum heart rate was recorded and used to calculate predicted maximum oxygen uptake adjusted for body weight (in milliliters per minute per kilogram) according to standard protocols. Sex-specific quartiles were formed based on performance at age 45 years. Study members in the lowest quartile were least fit. | (33) |
| Lung function (FEV1/FVC ratio post-test) | Pulmonary function was assessed by calculating post-albuterol (after 200 mg salbutamol) forced expiratory volume in one second (FEV1) and the ratio of FEV1 to forced vital capacity (FVC; FEV1/FVC) using measurements from spirometry conducted with a Sensormedics body plethysmograph (Sensormedics Corporation, Yorba Linda, CA, USA). Study members with an FEV1/FVC ratio below 0.70 were classified as having significant airflow limitation. | (34,35) |
| Total cholesterol | Serum non-fasting total cholesterol, triglycerides, and high-density lipoprotein (HDL) cholesterol levels (mmol/L) were measured by colorimetric assay on a Cobas c702 analyzer (Roche Diagnostics, Mannheim, Germany) at age 45 years. Sex-specific quartiles were formed based on total cholesterol levels at age 45 years. Study members in the highest quartile were considered to have high total cholesterol. | (36) |
| Triglycerides | Study members were considered to have an elevated triglyceride level if their value was 2.26 mmol/l or higher. | (36) |
| High-density lipoprotein (HDL) cholesterol | Study members were considered to have a low HDL cholesterol level if their value was 40mg/dL (1.04 mmol/L) or lower for men and 50 mg/dL (1.3 mmol/L) or lower for women. | (36) |
| Lipoprotein (a) | Serum lipoprotein(a) (nmol/L) was measured by a particle-enhanced immunoturbidimetric assay on a Cobas c502 analyzer (Roche Diagnostics, Mannheim, Germany) at age 45 years. Study members were considered to have a high Lipoprotein (a) level if their value ≥125 nmol/L or ≥50mg/dL. | (37) |
| ApoB100/ApoA1 | Serum apolipoprotein A1 and apolipoprotein B100 (g/L) were measured by immunoturbidimetric assay on a Cobas c502 analyzer (Roche Diagnostics, Mannheim, Germany), at age 45 years, and the ratio between the two was calculated. Sex-specific quartiles of ApoB100/ApoA1 were formed at age 45 years. Study members in the lowest quartile were least fit. | (38) |
| Estimated glomerular filtration rate (eGFR) | Serum creatinine (mmol/L) was measured by kinetic colorimetric assay on a Cobas c702 (Roche Diagnostics, Mannheim, Germany) at age 45 years. eGFR was estimated utilizing the CKD-Epi formula calculated from serum creatinine. Study members were considered to have a low eGFR level if their value was <90ml/min/1.73m^2^ (indicates stage G2 Poor-Normal). | (37,39) |
| Blood urea nitrogen (BUN) | Blood urea nitrogen (mmol/L) was measured by kinetic UV assay with Urease & Glutamate dehydrogenase assay on a Cobas c702 (Roche Diagnostics GmbH, D-68305 Mannheim, Germany). Sex-specific quartiles were formed based on BUN at age 45 years. Study members in the highest quartile were considered to have high BUN levels. |  |
| High-sensitivity C-reactive protein (hsCRP) | Elevation in inflammation was assessed by assaying High-sensitivity C-reactive protein (hsCRP, mg/L). High-sensitivity C-reactive protein level is thought to be one of the most reliable measured indicators of vascular inflammation and has been recently endorsed as an adjunct to traditional risk factor screening for cardiovascular risk. Serum C-reactive protein (mg/L) was measured by high sensitivity immunoturbidimetric assay on a Cobas c702 analyzer (Roche Diagnostics, Mannheim, Germany) at age 45 years. Study members were considered to have a high hsCRP level if their value was >3 mg/L. Untransformed hsCRP values greater than 10 mg/L were excluded to account for acute infection. | (40) |
| White blood cell count | Whole blood white blood cell counts (x10^9^cells/L) were measured by flow cytometry with a Sysmex XE5000 (Sysmex Corporation, Japan) at age 45 years. Study members were considered to have a high hsCRP level if their value was >11 x10^9^cells/L. | (41) |
| Pace of ageing (PoA) | As reported previously (16), the Pace of aging by age 45 years was quantified in three steps by assessing cardiovascular, metabolic, renal, immune, dental and pulmonary systems. First, longitudinal changes in 19 biomarkers were measured at ages 26, 32, 38 and 45 years. All biomarkers at each age were standardized on the basis of their original distribution at age 26 (that is, set to a mean of 0 and a standard deviation of 1). Second, linear mixed-effects modeling was used to quantify each study member’s personal rate of change across each of the 19 biomarkers. Third, we combined information from the 19 slopes to calculate each study member’s personal PoA, as the sum of age-dependent annual changes across all biomarkers. The resulting Pace of Aging was then scaled to a mean of 1 so that it could be interpreted with reference to an average rate of 1 year of biological aging per year of chronological aging (mean = 1, s.d. = 0.29).  The Pace of ageing by age 38 years used 18 biomarkers assayed at ages 26, 32 and 38, of which measures of leptin and caries-affected tooth surfaces, were not included. | (26,27) |

**Table S2.** Associations between age-5-years dmfs score and general health biomarkers among Dunedin study participants at 45 years of age, by a Poisson regression model using GEEs (variance robust).

|  | **Unadjusted** | | | |  | **Adjusted^b^** | | | |
| --- | --- | --- | --- | --- | --- | --- | --- | --- | --- |
|  | **IRR** | **IRR 95% CI** | **p** | **n** |  | **IRR** | **IRR 95% CI** | **p** | **n** |
| **Physical health biomarkers** | | |  |  |  |  |  |  |  |
| BMI | 1.02 | 1.01, 1.03 | 0.001 | 828 |  | 1.02 | 1.00, 1.03 | 0.006 | 825 |
| Waist-hip ratio | 1.02 | 1.00, 1.03 | 0.064 | 814 |  | 1.01 | 1.00, 1.03 | 0.107 | 811 |
| Waist circumference | 1.02 | 1.01, 1.03 | <0.001 | 815 |  | 1.02 | 1.01, 1.03 | <0.001 | 812 |
| HbA1c | 1.01 | 1.00, 1.03 | 0.059 | 789 |  | 1.01 | 1.00, 1.02 | 0.119 | 786 |
| Leptin | 1.02 | 1.01, 1.04 | 0.006 | 787 |  | 1.02 | 1.01, 1.04 | 0.008 | 784 |
| Mean arterial pressure | 1.00 | 0.98, 1.02 | 0.876 | 816 |  | 0.99 | 0.97, 1.01 | 0.272 | 813 |
| VO2max^a^ (rev) | 1.02 | 1.01, 1.04 | 0.009 | 768 |  | 1.00 | 0.99, 1.02 | 0.606 | 765 |
| FEV_1_/FVC^a^ | 0.97 | 0.93, 1.01 | 0.183 | 800 |  | 0.96 | 0.93, 1.01 | 0.087 | 796 |
| Total cholesterol | 1.02 | 1.01, 1.04 | 0.002 | 791 |  | 1.02 | 1.01, 1.04 | 0.006 | 788 |
| Triglycerides | 1.01 | 0.99, 1.02 | 0.269 | 791 |  | 1.00 | 0.99, 1.02 | 0.533 | 788 |
| HDL cholesterol | 1.01 | 0.99, 1.03 | 0.517 | 789 |  | 1.00 | 0.98, 1.03 | 0.705 | 786 |
| Lipoprotein (a) | 1.01 | 0.99, 1.03 | 0.411 | 790 |  | 1.01 | 0.99, 1.03 | 0.506 | 787 |
| ApoB/ApoA1 | 1.01 | 1.00, 1.03 | 0.108 | 786 |  | 1.01 | 1.00, 1.03 | 0.161 | 783 |
| eGFR | 0.99 | 0.97, 1.01 | 0.208 | 789 |  | 0.99 | 0.97, 1.01 | 0.289 | 786 |
| Blood urea nitrogen | 0.98 | 0.95, 1.01 | 0.113 | 791 |  | 0.98 | 0.96, 1.01 | 0.201 | 788 |
| hsCRP level | 1.02 | 1.00, 1.04 | 0.038 | 758 |  | 1.01 | 0.99, 1.03 | 0.157 | 755 |
| White blood cell count | 1.02 | 0.99, 1.06 | 0.234 | 789 |  | 1.02 | 0.98, 1.06 | 0.381 | 786 |

^a^ In the adjusted models, adjusted for sex, childhood SES, childhood IQ, perinatal health and BMI at age 45 years. ^b^ Adjusted for sex, childhood SES and childhood IQ and perinatal health. Abbreviations: OR = odds ratio, CI = confidence interval, BMI = body mass index, HbA1c = glycated haemoglobin, MAP = mean arterial pressure, VO_2_max = predicted maximum oxygen uptake adjusted for body weight in millilitres per minute per kilogram, (rev) = reverse, FEV_1_ = forced expiratory volume in one second, FVC = forced vital capacity, HDL = high-density lipoprotein levels, eGFR = estimated glomerular filtration rate, ApoB/ApoA1 = serum apolipoprotein B100/serum apolipoprotein A1 ratio, hsCRP = serum C-reactive protein.

**Table S3.** Associations between age-5-years dmft categories and general health biomarkers among Dunedin study participants at 45 years of age, by a Poisson regression model using GEEs (variance robust).

|  | **Unadjusted** | | | |  | **Adjusted^b^** | | | |
| --- | --- | --- | --- | --- | --- | --- | --- | --- | --- |
|  | **IRR** | **IRR 95% CI** | **p** | **n** |  | **IRR** | **IRR 95% CI** | **p** | **n** |
| **Physical health biomarkers^a^** | | |  |  |  |  |  |  |  |
| BMI |  |  |  | 828 |  |  |  |  | 825 |
| 1-4 dmft | 1.19 | 0.94, 1.49 | 0.141 |  |  | 1.15 | 0.91, 1.44 | 0.238 |  |
| 5+ dmft | 1.54 | 1.21, 1.97 | <0.001 |  |  | 1.47 | 1.16, 1.87 | 0.002 |  |
| Waist-hip ratio |  |  |  | 814 |  |  |  |  | 811 |
| 1-4 dmft | 1.38 | 1.06, 1.81 | 0.018 |  |  | 1.35 | 1.03, 1.78 | 0.028 |  |
| 5+ dmft | 1.37 | 0.99, 1.89 | 0.056 |  |  | 1.36 | 0.98, 1.88 | 0.066 |  |
| Waist circumference | |  |  | 815 |  |  |  |  | 812 |
| 1-4 dmft | 1.24 | 1.01, 1.52 | 0.045 |  |  | 1.19 | 0.97, 1.47 | 0.091 |  |
| 5+ dmft | 1.40 | 1.11, 1.77 | 0.005 |  |  | 1.34 | 1.07, 1.68 | 0.011 |  |
| HbA1c |  |  |  | 789 |  |  |  |  | 786 |
| 1-4 dmft | 0.99 | 0.80, 1.22 | 0.908 |  |  | 0.98 | 0.79, 1.20 | 0.826 |  |
| 5+ dmft | 1.21 | 0.96, 1.52 | 0.115 |  |  | 1.20 | 0.95, 1.52 | 0.120 |  |
| Leptin |  |  |  | 787 |  |  |  |  | 784 |
| 1-4 dmft | 1.10 | 0.83, 1.47 | 0.508 |  |  | 1.08 | 0.81, 1.44 | 0.606 |  |
| 5+ dmft | 1.48 | 1.09, 2.01 | 0.012 |  |  | 1.45 | 1.06, 1.98 | 0.018 |  |
| Mean arterial pressure | |  |  | 816 |  |  |  |  | 813 |
| 1-4 dmft | 1.21 | 0.94, 1.55 | 0.135 |  |  | 1.17 | 0.93, 1.48 | 0.182 |  |
| 5+ dmft | 0.99 | 0.71, 1.37 | 0.956 |  |  | 0.92 | 0.67, 1.25 | 0.577 |  |
| VO2max^c^ (rev) |  |  |  | 768 |  |  |  |  | 765 |
| 1-4 dmft | 1.11 | 0.83, 1.47 | 0.487 |  |  | 0.81 | 0.62, 1.06 | 0.129 |  |
| 5+ dmft | 1.43 | 1.05, 1.95 | 0.022 |  |  | 1.07 | 0.81, 1.41 | 0.624 |  |
| FEV_1_/FVC^c^ | |  |  | 800 |  |  |  |  | 796 |
| 1-4 dmft | 1.15 | 0.71, 1.84 | 0.573 |  |  | 1.14 | 0.71, 1.83 | 0.586 |  |
| 5+ dmft | 0.77 | 0.39, 1.49 | 0.431 |  |  | 0.73 | 0.37, 1.43 | 0.352 |  |
| Total cholesterol |  |  |  | 791 |  |  |  |  | 788 |
| 1-4 dmft | 0.87 | 0.64, 1.18 | 0.369 |  |  | 0.85 | 0.63, 1.15 | 0.301 |  |
| 5+ dmft | 1.37 | 1.01, 1.87 | 0.044 |  |  | 1.34 | 0.99, 1.83 | 0.058 |  |
| Triglycerides |  |  |  | 791 |  |  |  |  | 788 |
| 1-4 dmft | 1.17 | 0.94, 1.46 | 0.169 |  |  | 1.16 | 0.95, 1.41 | 0.155 |  |
| 5+ dmft | 1.12 | 0.85, 1.46 | 0.432 |  |  | 1.11 | 0.86, 1.45 | 0.422 |  |
| HDL cholesterol | |  |  | 789 |  |  |  |  | 786 |
| 1-4 dmft | 1.40 | 1.03, 1.92 | 0.034 |  |  | 1.36 | 1.00, 1.86 | 0.053 |  |
| 5+ dmft | 1.11 | 0.74, 1.66 | 0.606 |  |  | 1.09 | 0.72, 1.64 | 0.678 |  |
| Lipoprotein (a) |  |  |  | 790 |  |  |  |  | 787 |
| 1-4 dmft | 1.00 | 0.76, 1.32 | 0.984 |  |  | 0.98 | 0.74, 1.29 | 0.873 |  |
| 5+ dmft | 1.18 | 0.87, 1.61 | 0.286 |  |  | 1.16 | 0.85, 1.57 | 0.357 |  |
| ApoB/ApoA1 |  |  |  | 786 |  |  |  |  | 783 |
| 1-4 dmft | 1.17 | 0.88, 1.56 | 0.267 |  |  | 1.16 | 0.87, 1.54 | 0.318 |  |
| 5+ dmft | 1.35 | 0.98, 1.86 | 0.067 |  |  | 1.34 | 0.98, 1.85 | 0.070 |  |
| _e_GFR |  |  |  | 789 |  |  |  |  | 786 |
| 1-4 dmft | 0.84 | 0.69, 1.03 | 0.087 |  |  | 0.84 | 0.69, 1.02 | 0.080 |  |
| 5+ dmft | 0.84 | 0.65, 1.07 | 0.161 |  |  | 0.85 | 0.66, 1.09 | 0.190 |  |
| Blood urea nitrogen | |  |  | 791 |  |  |  |  | 788 |
| 1-4 dmft | 0.83 | 0.62, 1.10 | 0.193 |  |  | 0.84 | 0.63, 1.11 | 0.220 |  |
| 5+ dmft | 0.89 | 0.63, 1.25 | 0.491 |  |  | 0.94 | 0.66, 1.32 | 0.707 |  |
| hsCRP |  |  |  | 758 |  |  |  |  | 755 |
| 1-4 dmft | 1.02 | 0.73, 1.42 | 0.930 |  |  | 0.98 | 0.70, 1.37 | 0.907 |  |
| 5+ dmft | 1.50 | 1.05, 2.13 | 0.025 |  |  | 1.40 | 0.99, 1.98 | 0.059 |  |
| White blood cell count | |  |  | 789 |  |  |  |  | 786 |
| 1-4 dmft | 1.12 | 0.54, 2.35 | 0.760 |  |  | 1.07 | 0.51, 2.26 | 0.849 |  |
| 5+ dmft | 1.72 | 0.79, 3.76 | 0.171 |  |  | 1.64 | 0.75, 3.58 | 0.217 |  |

^a^Comparison group = Caries-free (dmft=0), ^b^for sex, childhood SES, childhood IQ and perinatal health, ^c^ adjusted for sex, childhood SES, childhood IQ, perinatal health and BMI at age 45 years. Abbreviations: CI = confidence interval, BMI = body mass index, VO_2_Max = predicted maximum oxygen uptake adjusted for body weight in millilitres per minute per kilogram, (rev) = reverse, FEV_1_ = forced expiratory volume in one second, FVC = forced vital capacity, HDL = high-density lipoprotein levels, ApoB/ApoA1 = serum apolipoprotein B100/serum apolipoprotein A1 ratio, eGFR = estimated glomerular filtration rate, hsCRP = serum C-reactive protein.

**Table S4.** Associations between age-5-years dmft score and general health biomarkers among Dunedin study participants at 45 years of age, by a Poisson regression model using GEEs (variance robust).

|  | **Unadjusted** | | | |  | **Adjusted^b^** | | | |
| --- | --- | --- | --- | --- | --- | --- | --- | --- | --- |
|  | **IRR** | **IRR 95% CI** | **p** | **n** |  | **IRR** | **IRR 95% CI** | **p** | **n** |
| **Physical health biomarkers** | | |  |  |  |  |  |  |  |
| BMI | 1.05 | 1.02, 1.08 | <0.001 | 828 |  | 1.04 | 1.02, 1.07 | 0.001 | 825 |
| Waist-hip ratio | 1.04 | 1.00, 1.07 | 0.028 | 814 |  | 1.04 | 1.00, 1.07 | 0.043 | 811 |
| Waist circumference | 1.05 | 1.02, 1.07 | <0.001 | 815 |  | 1.04 | 1.02, 1.06 | <0.001 | 812 |
| HbA1c | 1.03 | 1.00, 1.05 | 0.050 | 789 |  | 1.03 | 1.00, 1.05 | 0.063 | 786 |
| Leptin | 1.05 | 1.02, 1.08 | 0.004 | 787 |  | 1.05 | 1.01, 1.08 | 0.005 | 784 |
| Mean arterial pressure | 0.99 | 0.96, 1.03 | 0.616 | 816 |  | 0.98 | 0.95, 1.01 | 0.244 | 813 |
| VO2max^a^ (rev) | 1.05 | 1.01, 1.08 | 0.007 | 768 |  | 1.01 | 0.98, 1.04 | 0.532 | 765 |
| FEV_1_/FVC^a^ | 0.95 | 0.89, 1.02 | 0.158 | 800 |  | 0.94 | 0.88, 1.01 | 0.095 | 796 |
| Total cholesterol | 1.05 | 1.02, 1.09 | 0.004 | 791 |  | 1.05 | 1.01, 1.08 | 0.007 | 788 |
| Triglycerides | 1.02 | 0.99, 1.05 | 0.206 | 791 |  | 1.02 | 0.99, 1.05 | 0.236 | 788 |
| HDL cholesterol | 1.03 | 0.99, 1.07 | 0.189 | 789 |  | 1.02 | 0.98, 1.07 | 0.278 | 786 |
| Lipoprotein (a) | 1.01 | 0.97, 1.05 | 0.584 | 790 |  | 1.01 | 0.97, 1.05 | 0.666 | 787 |
| ApoB/ApoA1 | 1.04 | 1.01, 1.08 | 0.019 | 786 |  | 1.04 | 1.00, 1.08 | 0.029 | 783 |
| eGFR | 0.97 | 0.94, 1.01 | 0.126 | 789 |  | 0.98 | 0.95, 1.01 | 0.168 | 786 |
| Blood urea nitrogen | 0.96 | 0.92, 1.01 | 0.091 | 791 |  | 0.97 | 0.93, 1.01 | 0.179 | 788 |
| hsCRP level | 1.04 | 1.00, 1.08 | 0.044 | 758 |  | 1.03 | 0.99, 1.07 | 0.145 | 755 |
| White blood cell count | 1.06 | 0.98, 1.15 | 0.172 | 789 |  | 1.05 | 0.97, 1.14 | 0.262 | 786 |

^a^ In the adjusted models, adjusted for sex, childhood SES, childhood IQ, perinatal health and BMI at age 45 years. ^b^ Adjusted for sex, childhood SES, childhood IQ and perinatal health. Abbreviations: OR = odds ratio, CI = confidence interval, BMI = body mass index, HbA1c = glycated haemoglobin, MAP = mean arterial pressure, VO_2_max = predicted maximum oxygen uptake adjusted for body weight in millilitres per minute per kilogram, FEV_1_ = forced expiratory volume in one second, FVC = forced vital capacity, HDL = high-density lipoprotein levels, eGFR = estimated glomerular filtration rate, ApoB/ApoA1 = serum apolipoprotein B100/serum apolipoprotein A1 ratio, hsCRP = serum C-reactive protein.

**Table S5.** Associations between age-5-years caries experience, ‘any caries’ (dmft 1+ teeth) and general health biomarkers among Dunedin study participants at 45 years of age, by a Poisson regression model using GEEs (variance robust).

|  | **Unadjusted** | | | |  | **Adjusted^b^** | | | |
| --- | --- | --- | --- | --- | --- | --- | --- | --- | --- |
|  | **IRR** | **IRR 95% CI** | **p** | **n** |  | **IRR** | **IRR 95% CI** | **p** | **n** |
| **Physical health biomarkers^a^** | | |  |  |  |  |  |  |  |
| BMI | 1.31 | 1.07, 1.60 | 0.010 | 828 |  | 1.26 | 1.02, 1.54 | 0.028 | 825 |
| Waist-hip ratio | 1.38 | 1.07, 1.77 | 0.012 | 814 |  | 1.35 | 1.05, 1.74 | 0.019 | 811 |
| Waist circumference | 1.29 | 1.07, 1.56 | 0.008 | 815 |  | 1.24 | 1.03, 1.50 | 0.022 | 812 |
| HbA1c | 1.06 | 0.88, 1.28 | 0.533 | 789 |  | 1.05 | 0.87, 1.26 | 0.593 | 786 |
| Leptin | 1.23 | 0.95, 1.59 | 0.113 | 787 |  | 1.20 | 0.93, 1.56 | 0.160 | 784 |
| MAP | 1.14 | 0.90, 1.43 | 0.284 | 816 |  | 1.08 | 0.87, 1.35 | 0.469 | 813 |
| VO2Max^c^ (rev) | 1.22 | 0.94, 1.57 | 0.129 | 768 |  | 0.91 | 0.72, 1.14 | 0.417 | 765 |
| FEV_1_/FVC^c^ | 1.02 | 0.65, 1.59 | 0.941 | 800 |  | 1.00 | 0.64, 1.57 | 0.998 | 796 |
| Total cholesterol | 1.04 | 0.80, 1.35 | 0.767 | 791 |  | 1.02 | 0.78, 1.32 | 0.899 | 788 |
| Triglycerides | 1.15 | 0.94, 1.41 | 0.181 | 791 |  | 1.14 | 0.95, 1.38 | 0.163 | 788 |
| HDL cholesterol | 1.30 | 0.97, 1.75 | 0.077 | 789 |  | 1.27 | 0.94, 1.71 | 0.113 | 786 |
| Lipoprotein (a) | 1.06 | 0.83, 1.36 | 0.620 | 790 |  | 1.04 | 0.81, 1.32 | 0.771 | 787 |
| ApoB/ApoA1 | 1.23 | 0.95, 1.60 | 0.110 | 786 |  | 1.22 | 0.94, 1.58 | 0.133 | 783 |
| eGFR | 0.84 | 0.70, 1.00 | 0.054 | 789 |  | 0.84 | 0.70, 1.00 | 0.056 | 786 |
| Blood urea nitrogen | 0.85 | 0.66, 1.09 | 0.202 | 791 |  | 0.87 | 0.67, 1.12 | 0.280 | 788 |
| hsCRP | 1.18 | 0.88, 1.58 | 0.275 | 758 |  | 1.13 | 0.84, 1.51 | 0.428 | 755 |
| White blood cell count | 1.33 | 0.69, 2.55 | 0.399 | 789 |  | 1.26 | 0.65, 2.45 | 0.488 | 786 |

^a^Comparison group = Caries-free (dmft=0), ^b^Adjusted for sex, childhood SES, childhood IQ and perinatal health. ^c^Adjusted for sex, childhood SES, childhood IQ, perinatal health and BMI at age 45 years. Abbreviations: CI = confidence interval, BMI = body mass index, VO_2_Max = predicted maximum oxygen uptake adjusted for body weight in millilitres per minute per kilogram, (rev) = reverse, FEV_1_ = forced expiratory volume in one second, FVC = forced vital capacity, HDL = high-density lipoprotein levels, ApoB/ApoA1 = serum apolipoprotein B100/serum apolipoprotein A1 ratio, eGFR = estimated glomerular filtration rate, hsCRP = serum C-reactive protein.

**
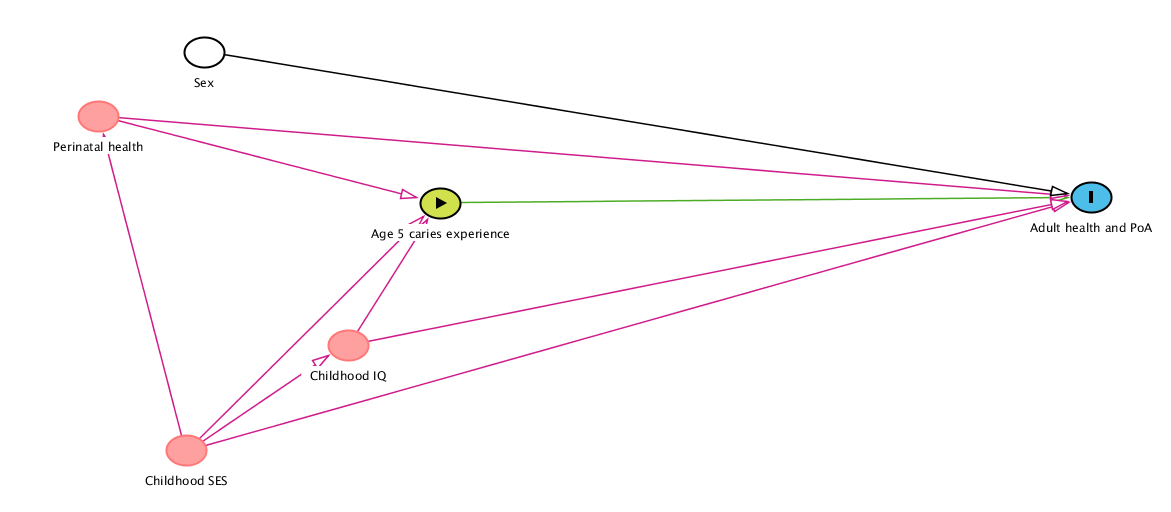
**

**Figure S1.** Directed acyclic graph conceptualising the study’s analytical approach.

**
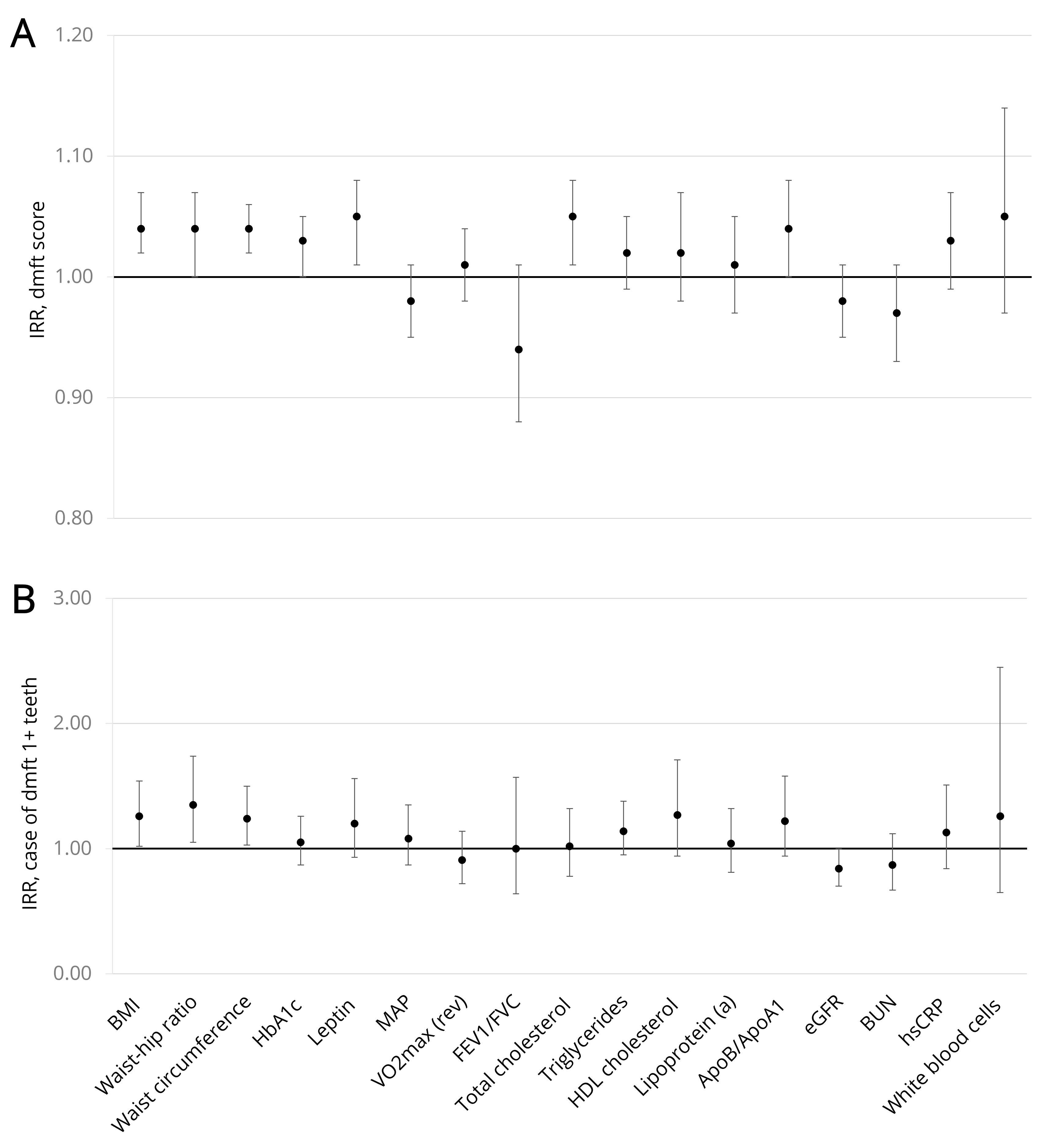
**

**Figure S2.** Associations between age-5y caries experience and general health biomarkers at age 45 years among Dunedin study participants. **A,** Poisson regression model for biomarker thresholds by dmft score at age 5 years. **B,** Logistic regression model for biomarker thresholds by ‘case of dmft +1’ at age 5 years, comparison group = caries-free.

**Table S6.** Associations between age-5-years dmfs and general health biomarkers among Dunedin study participants at 45 years of age, using quantile regression (median regression, variance robust).

|  | Unadjusted | | | |  | Adjusted for sex, childhood SES and childhood IQ and perinatal health | | | |
| --- | --- | --- | --- | --- | --- | --- | --- | --- | --- |
|  | β | β 95% CI | p | n |  | β | β 95% CI | p | n |
| **Physical health biomarkers** | | |  |  |  |  |  |  |  |
| BMI | 0.182 | 0.097, 0.268 | <0.001 | 828 |  | 0.153 | 0.052, 0.254 | 0.003 | 825 |
| Waist-hip ratio | 0.001 | -0.000, 0.002 | 0.098 | 814 |  | 0.000 | -0.001, 0.001 | 0.984 | 811 |
| Waist circumference | 0.417 | 0.266, 0.567 | <0.001 | 815 |  | 0.271 | 0.082, 0.460 | 0.005 | 812 |
| Hemoglobin A1c | 0.004 | -0.000, 0.008 | 0.074 | 789 |  | 0.001 | -0.003, 0.004 | 0.585 | 786 |
| Leptin | 0.260 | -0.108, 0.629 | 0.165 | 787 |  | 0.137 | -0.057, 0.330 | 0.166 | 784 |
| Mean arterial pressure | 0.035 | -0.131, 0.201 | 0.677 | 816 |  | -0.050 | -0.170, 0.070 | 0.412 | 813 |
| VO2Max ^a^ | -0.042 | -0.193, 0.109 | 0.582 | 768 |  | -0.004 | -0.062, 0.054 | 0.894 | 765 |
| FEV1/FVC post-test ^a^ | 0.087 | 0.033, 0.142 | 0.002 | 800 |  | 0.093 | 0.028, 0.159 | 0.005 | 796 |
| Total cholesterol | 0.018 | 0.001, 0.035 | 0.042 | 791 |  | 0.011 | -0.003, 0.024 | 0.127 | 788 |
| Triglycerides | 0.009 | -0.006, 0.024 | 0.246 | 791 |  | 0.007 | -0.007, 0.022 | 0.321 | 788 |
| HDL cholesterol | -0.004 | -0.009, 0.002 | 0.229 | 789 |  | -0.003 | -0.008, 0.002 | 0.197 | 786 |
| Lipoprotein (a) | 1.238 | -2.474, 4.950 | 0.513 | 790 |  | 1.574 | -2.034, 5.182 | 0.392 | 787 |
| ApoB/ApoA1 | 0.005 | 0.002, 0.007 | <0.001 | 786 |  | 0.002 | -0.001, 0.005 | 0.113 | 783 |
| eGFR ^b^ | 0.139 | -0.030,0.308 | 0.106 | 789 |  | 0.079 | -0.080, 0.238 | 0.331 | 0.079 |
| Blood urea nitrogen | -0.002 | -0.016, 0.012 | 0.764 | 791 |  | -0.001 | -0.016, 0.014 | 0.923 | 788 |
| hsCRP | 0.029 | -0.001, 0.059 | 0.056 | 758 |  | 0.007 | -0.015, 0.029 | 0.543 | 755 |
| White blood cell count | 0.012 | -0.009, 0.034 | 0.263 | 789 |  | 0.020 | -0.005, 0.044 | 0.111 | 786 |

^a^ In the adjusted models, adjusted for sex, childhood SES, childhood IQ, perinatal health and BMI at age 45 years. ^b^ Unadjusted and adjusted models represent quantile regressions for the 25^th^ percentile. Abbreviations: CI = confidence interval, BMI = body mass index, VO_2_Max = predicted maximum oxygen uptake adjusted for body weight in milliliters per minute per kilogram, FEV_1_ = forced expiratory volume in one second, FVC = forced vital capacity, HDL = high-density lipoprotein levels, eGFR = estimated glomerular filtration rate, ApoB/ApoA1 = serum apolipoprotein B100/serum apolipoprotein A1 ratio, hsCRP = serum C-reactive protein.

**Table S7.** Associations between age-5-years dmft categories and general health biomarkers among Dunedin study participants at 45 years of age, using quantile regression (median regression, variance robust).

|  | Unadjusted | | | |  | Adjusted for sex, childhood SES, childhood IQ  and perinatal health | | | |
| --- | --- | --- | --- | --- | --- | --- | --- | --- | --- |
|  | β | 95% CI | p | n |  | β | 95% CI | p | n |
| **Physical health biomarkers ^a^** | |  |  |  |  |  |  |  |  |
| BMI |  |  |  | 828 |  |  |  |  | 825 |
| dmft 1-4 | 1.221 | 0.261, 2.181 | 0.013 |  |  | 0.858 | -0.052, 1.767 | 0.065 |  |
| dmft 5+ | 2.712 | 1.682, 3.743 | <0.001 |  |  | 2.270 | 1.323, 3.216 | <0.001 |  |
| Waist-hip ratio |  |  |  | 814 |  |  |  |  | 811 |
| dmft 1-4 | 0.015 | -0.001, 0.031 | 0.075 |  |  | 0.015 | 0.004, 0.027 | 0.008 |  |
| dmft 5+ | 0.019 | 0.001, 0.038 | 0.038 |  |  | 0.004 | -0.010, 0.018 | 0.589 |  |
| Waist circumference |  |  |  | 815 |  |  |  |  | 812 |
| dmft 1-4 | 3.150 | 0.387, 5.913 | 0.025 |  |  | 1.346 | -1.253, 3.945 | 0.310 |  |
| dmft 5+ | 5.450 | 2.435, 8.465 | <0.001 |  |  | 3.200 | 0.255, 6.145 | 0.033 |  |
| Hemoglobin A1c ^b^ |  |  |  | 789 |  |  |  |  | 786 |
| dmft 1-4 | 0.091 | 0.024, 0.159 | 0.008 |  |  | 0.006 | -0.042, 0.054 | 0.798 |  |
| dmft 5+ | 0.091 | 0.001, 0.181 | 0.046 |  |  | 0.039 | -0.009, 0.087 | 0.108 |  |
| Leptin |  |  |  | 787 |  |  |  |  | 784 |
| dmft 1-4 | 1.608 | -1.335, 4.551 | 0.284 |  |  | 1.037 | -0.475, 2.548 | 0.179 |  |
| dmft 5+ | 3.667 | -0.763, 8.098 | 0.105 |  |  | 2.874 | 0.440, 5.309 | 0.021 |  |
| Mean arterial pressure | |  |  | 816 |  |  |  |  | 813 |
| dmft 1-4 | 0.107 | -2.250, 2.465 | 0.929 |  |  | -0.973 | -2.864, 0.917 | 0.313 |  |
| dmft 5+ | -0.540 | -3.525, 2.445 | 0.723 |  |  | -1.425 | -3.423, 0.574 | 0.162 |  |
| VO_2_Max ^c^ |  |  |  | 768 |  |  |  |  | 765 |
| dmft 1-4 | -0.546 | -2.202, 1.109 | 0.517 |  |  | -0.218 | -1.036, 0.601 | 0.602 |  |
| dmft 5+ | -1.313 | -3.567, 0.942 | 0.253 |  |  | -0.519 | -1.392, 0.354 | 0.244 |  |
| FEV_1_/FVC post-test ^c^ | |  |  | 800 |  |  |  |  | 796 |
| dmft 1-4 | 0.058 | -1.093, 1.209 | 0.922 |  |  | 0.022 | -0.924, 0.968 | 0.963 |  |
| dmft 5+ | 0.874 | -0.424, 2.171 | 0.187 |  |  | 0.746 | -0.379, 1.871 | 0.193 |  |
| Total cholesterol |  |  |  | 791 |  |  |  |  | 788 |
| dmft 1-4 | -0.100 | -0.305, 0.105 | 0.338 |  |  | -0.077 | -0.255, 0.101 | 0.395 |  |
| dmft 5+ | 0.200 | -0.072, 0.472 | 0.149 |  |  | 0.180 | -0.056, 0.417 | 0.134 |  |
| Triglycerides |  |  |  | 791 |  |  |  |  | 788 |
| dmft 1-4 | 0.200 | -0.006, 0.406 | 0.056 |  |  | 0.128 | -0.027, 0.282 | 0.106 |  |
| dmft 5+ | 0.200 | -0.058, 0.458 | 0.129 |  |  | 0.099 | -0.077, 0.275 | 0.271 |  |
| HDL cholesterol |  |  |  | 789 |  |  |  |  | 786 |
| dmft 1-4 | -0.101 | -0.182, -0.020 | 0.015 |  |  | -0.068 | -0.141, 0.005 | 0.066 |  |
| dmft 5+ | -0.045 | -0.144, 0.054 | 0.371 |  |  | -0.046 | -0.136, 0.044 | 0.317 |  |
| Lipoprotein (a) |  |  |  | 790 |  |  |  |  | 787 |
| dmft 1-4 | -11.867 | -48.995, 25.262 | 0.531 |  |  | -6.826 | -44.410, 30.757 | 0.722 |  |
| dmft 5+ | -12.032 | -72.392, 48.328 | 0.696 |  |  | 1.792 | -49.497, 53.081 | 0.945 |  |
| ApoB/ApoA1 |  |  |  | 786 |  |  |  |  | 783 |
| dmft 1-4 | 0.022 | -0.020, 0.064 | 0.310 |  |  | 0.009 | -0.020, 0.038 | 0.559 |  |
| dmft 5+ | 0.047 | -0.002, 0.096 | 0.058 |  |  | 0.042 | 0.001, 0.083 | 0.047 |  |
| _e_GFR ^d^ |  |  |  | 789 |  |  |  |  | 786 |
| dmft 1-4 | 3.000 | 0.676, 5.324 | 0.011 |  |  | 2.891 | 0.849, 4.932 | 0.006 |  |
| dmft 5+ | 3.000 | -0.634, 6.634 | 0.106 |  |  | 2.861 | -0.171, 5.892 | 0.064 |  |
| Blood urea nitrogen |  |  |  | 791 |  |  |  |  | 788 |
| dmft 1-4 | -0.247 | -0.481, -0.013 | 0.039 |  |  | -0.093 | -0.282, 0.096 | 0.335 |  |
| dmft 5+ | -0.110 | -0.334, 0.114 | 0.336 |  |  | -0.053 | -0.311, 0.205 | 0.687 |  |
| hsCRP |  |  |  | 758 |  |  |  |  | 755 |
| dmft 1-4 | 0.150 | -0.089, 0.389 | 0.219 |  |  | 0.006 | -0.207, 0.219 | 0.953 |  |
| dmft 5+ | 0.410 | -0.076, 0.896 | 0.098 |  |  | 0.324 | -0.120, 0.768 | 0.152 |  |
| White blood cell count | |  |  | 789 |  |  |  |  | 786 |
| dmft 1-4 | 0.200 | -0.097, 0.497 | 0.187 |  |  | 0.234 | -0.077, 0.546 | 0.140 |  |
| dmft 5+ | 0.400 | 0.037, 0.763 | 0.031 |  |  | 0.243 | -0.186, 0.672 | 0.266 |  |

^a^ Comparison group = Caries-free (dmft=0), ^b^ Unadjusted model represents quantile regression for the 75^th^ percentile, ^c^ In the adjusted models, adjusted for sex, childhood SES, childhood IQ, perinatal health and BMI at age 45 years, ^d^ Quantile regression for the 25^th^ percentile. Abbreviations: CI = confidence interval, BMI = body mass index, VO_2_Max = predicted maximum oxygen uptake adjusted for body weight in milliliters per minute per kilogram, FEV_1_ = forced expiratory volume in one second, FVC = forced vital capacity, HDL = high-density lipoprotein levels, ApoB/ApoA1 = serum apolipoprotein B100/serum apolipoprotein A1 ratio, eGFR = estimated glomerular filtration rate, hsCRP = serum C-reactive protein.

**Table S8.** Associations between age-5-years dmft and general health biomarkers among Dunedin study participants at 45 years of age, using quantile regression (median regression, variance robust).

|  | Unadjusted | | | |  | Adjusted for sex, childhood SES and childhood IQ and perinatal health | | | |
| --- | --- | --- | --- | --- | --- | --- | --- | --- | --- |
|  | β | β 95% CI | p | n |  | β | β 95% CI | p | n |
| **Physical health biomarkers** | | |  |  |  |  |  |  |  |
| BMI | 0.323 | 0.175, 0.471 | <0.001 | 828 |  | 0.255 | 0.113, 0.396 | <0.001 | 825 |
| Waist-hip ratio | 0.002 | 0.000, 0.005 | 0.037 | 814 |  | 0.000 | -0.001, 0.002 | 0.781 | 811 |
| Waist circumference | 0.833 | 0.518, 1.149 | <0.001 | 815 |  | 0.629 | 0.282, 0.976 | <0.001 | 812 |
| HbA1c | 0.011 | 0.004, 0.019 | 0.004 | 789 |  | 0.003 | -0.004, 0.011 | 0.365 | 786 |
| Leptin | 0.680 | 0.110, 1.250 | 0.019 | 787 |  | 0.342 | 0.029, 0.654 | 0.032 | 784 |
| Mean arterial pressure | 0.054 | -0.265, 0.374 | 0.738 | 816 |  | -0.126 | -0.352, 0.100 | 0.275 | 813 |
| VO2max ^a^ (rev) | -0.178 | -0.436, 0.080 | 0.176 | 768 |  | -0.050 | -0.136, 0.037 | 0.258 | 765 |
| FEV1/FVC ^a^ | 0.180 | 0.044, 0.317 | 0.010 | 800 |  | 0.176 | 0.031, 0.322 | 0.018 | 796 |
| Total cholesterol | 0.040 | 0.009, 0.071 | 0.012 | 791 |  | 0.022 | -0.007, 0.051 | 0.131 | 788 |
| Triglycerides | 0.023 | -0.005, 0.052 | 0.112 | 791 |  | 0.015 | -0.009, 0.039 | 0.220 | 788 |
| HDL cholesterol | -0.006 | -0.018, 0.006 | 0.329 | 789 |  | -0.008 | -0.018, 0.002 | 0.128 | 786 |
| Lipoprotein (a) | -0.782 | -7.072, 5.509 | 0.807 | 790 |  | 1.388 | -5.015, 7.792 | 0.670 | 787 |
| ApoB/ApoA1 | 0.009 | 0.003, 0.014 | 0.003 | 786 |  | 0.005 | 0.000, 0.010 | 0.032 | 783 |
| eGFR ^b^ | 0.334 | -0.002, 0.670 | 0.051 | 789 |  | 0.307 | 0.100, 0.514 | 0.004 | 786 |
| Blood urea nitrogen | -0.011 | -0.041, 0.018 | 0.449 | 791 |  | -0.010 | -0.039, 0.019 | 0.512 | 788 |
| hsCRP level | 0.065 | 0.019, 0.111 | 0.006 | 758 |  | 0.028 | -0.012, 0.067 | 0.165 | 755 |
| White blood cell count | 0.050 | 0.004, 0.096 | 0.032 | 789 |  | 0.037 | -0.011, 0.084 | 0.133 | 786 |

^a^ In the adjusted models, adjusted for sex, childhood SES, childhood IQ, perinatal health and BMI at age 45 years, ^b^ Unadjusted and adjusted models represent quantile regression for the 25^th^ percentile. Abbreviations: OR = odds ratio, CI = confidence interval, BMI = body mass index, HbA1c = glycated haemoglobin, MAP = mean arterial pressure, VO_2_max = predicted maximum oxygen uptake adjusted for body weight in milliliters per minute per kilogram, FEV_1_ = forced expiratory volume in one second, FVC = forced vital capacity, HDL = high-density lipoprotein levels, eGFR = estimated glomerular filtration rate, ApoB/ApoA1 = serum apolipoprotein B100/serum apolipoprotein A1 ratio, hsCRP = serum C-reactive protein.

**Table S9.** Associations between age-5-years caries experience, ‘any caries’ (dmft 1+ teeth) and general health biomarkers among Dunedin study participants at 45 years of age, using quantile regression (median regression, variance robust).

|  | Unadjusted | | | |  | Adjusted for sex, childhood SES, childhood IQ and perinatal health | | | |
| --- | --- | --- | --- | --- | --- | --- | --- | --- | --- |
|  | β | β 95% CI | p | n |  | β | β 95% CI | p | n |
| **Physical health biomarkers ^a^** | | |  |  |  |  |  |  |  |
| BMI | 1.797 | 0.940, 2.653 | <0.001 | 828 |  | 1.351 | 0.576, 2.125 | 0.001 | 825 |
| Waist-hip ratio | 0.016 | 0.001, 0.030 | 0.032 | 814 |  | 0.013 | 0.003, 0.024 | 0.011 | 811 |
| Waist circumference | 3.900 | 1.293, 6.507 | 0.003 | 815 |  | 2.050 | -0.261,4.361 | 0.082 | 812 |
| Hemoglobin A1c | 0.091 | 0.061, 0.122 | <0.001 | 789 |  | 0.020 | -0.022, 0.062 | 0.352 | 786 |
| Leptin | 2.523 | -0.221, 5.267 | 0.071 | 787 |  | 1.317 | -0.122, 2.755 | 0.073 | 784 |
| Mean arterial pressure | 0.009 | -2.129, 2.147 | 0.994 | 816 |  | -1.194 | -2.842, 0.454 | 0.155 | 813 |
| VO2Max ^b^ | -0.823 | -2.330, 0.684 | 0.284 | 768 |  | -0.235 | -0.935, 0.465 | 0.510 | 765 |
| FEV1/FVC post-test ^b^ | 0.239 | -0.781, 1.259 | 0.646 | 800 |  | 0.303 | -0.527, 1.134 | 0.474 | 796 |
| Total cholesterol ^c^ | 0.100 | -0.095, 0.295 | 0.315 | 791 |  | -0.009 | -0.166, 0.149 | 0.916 | 788 |
| Triglycerides | 0.200 | 0.016, 0.384 | 0.033 | 791 |  | 0.097 | -0.038, 0.231 | 0.159 | 788 |
| HDL cholesterol | -0.085 | -0.163, -0.007 | 0.032 | 789 |  | -0.065 | -0.130, -0.001 | 0.046 | 786 |
| Lipoprotein (a) | -12.032 | -46.930, 22.866 | 0.499 | 790 |  | -5.618 | -39.447, 28.211 | 0.745 | 787 |
| ApoB/ApoA1 | 0.034 | -0.003, 0.071 | 0.069 | 786 |  | 0.023 | -0.004, 0.050 | 0.101 | 783 |
| eGFR ^d^ | 3.000 | 0.886, 5.114 | 0.005 | 789 |  | 2.892 | 0.880, 4.904 | 0.005 | 786 |
| Blood urea nitrogen | -0.220 | -0.414, -0.025 | 0.027 | 791 |  | -0.079 | -0.257, 0.099 | 0.383 | 788 |
| hsCRP | 0.220 | -0.013, 0.453 | 0.064 | 758 |  | 0.044 | -0.165, 0.253 | 0.679 | 755 |
| White blood cell count | 0.300 | 0.030, 0.570 | 0.030 | 789 |  | 0.242 | -0.033, 0.517 | 0.085 | 786 |

^a^ Comparison group = Caries-free (dmft=0), ^b^ In the adjusted models adjusted for sex, childhood SES, childhood IQ, perinatal health and BMI at age 45 years, ^c^ Unadjusted model represents quantile regression for the 25^th^ percentile, ^d^ Unadjusted and adjusted models represent quantile regressions for the 25^th^ percentile. Abbreviations: CI = confidence interval, BMI = body mass index, VO_2_Max = predicted maximum oxygen uptake adjusted for body weight in milliliters per minute per kilogram, FEV_1_ = forced expiratory volume in one second, FVC = forced vital capacity, HDL = high-density lipoprotein levels, ApoB/ApoA1 = serum apolipoprotein B100/serum apolipoprotein A1 ratio, eGFR = estimated glomerular filtration rate, hsCRP = serum C-reactive protein.

**Table S10.** Associations between caries experience at age-5-years and the Pace of Ageing among Dunedin study participants at 38 years of age.

|  | Unadjusted (n=856) | | |  | Adjusted for sex, childhood SES and childhood IQ and perinatal health (n=853) | | |
| --- | --- | --- | --- | --- | --- | --- | --- |
|  | β | β 95% CI | p |  | β | β 95% CI | p |
| **Caries experience at age 5 years** | | |  |  |  |  |  |
| dmfs | 0.005 | -0.001, 0.010 | 0.078 |  | 0.002 | -0.003, 0.007 | 0.374 |
| dmft | 0.010 | 0.002, 0.018 | 0.017 |  | 0.007 | -0.002, 0.015 | 0.128 |
| Caries-free | Ref. |  |  |  |  |  |  |
| dmft 1-4 | 0.068 | 0.015, 0.121 | 0.012 |  | 0.055 | 0.003, 0.107 | 0.037 |
| dmft 5+ | 0.109 | 0.043, 0.175 | 0.001 |  | 0.086 | 0.019, 0.153 | 0.011 |
| Caries-free | Ref. |  |  |  |  |  |  |
| Any caries | 0.082 | 0.035, 0.130 | 0.001 |  | 0.066 | 0.019, 0.112 | 0.006 |

Abbreviations: β = ordinary least squares regression beta coefficients, CI = confidence interval, Ref. = reference category (comparison group = dmft=0).

**References**

1. Poulton R, Moffitt TE, Silva PA. The Dunedin multidisciplinary health and development study: Overview of the first 40 years, with an eye to the future. Soc Psychiatry Psychiatr Epidemiol. 2015 May;50(5):679–93.

2. Poulton R, Guiney H, Ramrakha S, Moffitt TE. The Dunedin study after half a century: reflections on the past, and course for the future. J R Soc New Zeal. 2022;DOI: 10.1080/03036758.2022.2114508.

3. Poulton R, Robertson K, Boden J, Horwood J, Theodore R, Potiki T, et al. Patterns of recreational cannabis use in Aotearoa-New Zealand and their consequences: Evidence to inform voters in the 2020 referendum. J R Soc New Zeal. 2020 Apr 2;50(2):348–65.

4. World Health Organization. Oral health surveys: Basic methods. Second ed. Geneva: World Health Organization; 1977.

5. Klein H, Palmer CE, Knutson JW. Studies on dental caries: I. Dental status and dental needs of elementary school children. Public Heal Reports. 1938;53(19):751–65.

6. Broadbent JM, Ayers KMS, Thomson WM. Is attention-deficit hyperactivity disorder a risk factor for dental caries? A case-control study. Caries Res. 2004;38:29–33.

7. Evans RW, Beck DJ, Brown RH. Dental health of 5-year-old children: A report from the Dunedin multidisciplinary child development study. N Z Dent J. 1980 Oct;76(346):179–86.

8. Evans RW, Beck DJ, Silva PA, Brown RH. Relationships between dental health behaviour and oral health status of 5-year-old children: A report from the Dunedin multidisciplinary child development study. N Z Dent J. 1982 Jan;78(351):11–6.

9. Evans RW, Beck DJ, Brown RH, Silva PA. Relationship between fluoridation and socioeconomic status on dental caries experience in 5-year-old New Zealand children. Community Dent Oral Epidemiol. 1984;12(1):5–9.

10. Poulton R, Caspi A, Milne BJ, Thomson WM, Taylor A, Sears MR, et al. Association between children’s experience of socioeconomic disadvantage and adult health: A life-course study. Lancet. 2002 Nov 23;360(9346):1640–5.

11. Ministry of Health. Our oral health: Key findings of the 2009 New Zealand oral health survey. Wellington; 2010.

12. Shalev I, Caspi A, Ambler A, Belsky DW, Chapple S, Cohen HJ, et al. Perinatal complications and aging indicators by midlife. Pediatrics. 2014 Nov;134(5):e1315–23.

13. Ben-Shlomo Y, Kuh D. A life course approach to chronic disease epidemiology: Conceptual models, empirical challenges and interdisciplinary perspectives. Int J Epidemiol. 2002 Apr 1;31(2):285–93.

14. Marmot M. Fair Society, Healthy Lives: The Marmot Review; Strategic Review of Health Inequalities in England post-2010. London: Institute of Health Equity; 2010.

15. Sabbah W, Tsakos G, Chandola T, Sheiham A, Watt RG. Social gradients in oral and general health. J Dent Res. 2007 Oct;86(10):992–6.

16. Thomson WM. Social inequality in oral health. Community Dent Oral Epidemiol. 2012 Oct;40:28–32.

17. Thomson WM, Poulton R, Milne BJ, Caspi A, Broughton JR, Ayers KMS. Socioeconomic inequalities in oral health in childhood and adulthood in a birth cohort. Community Dent Oral Epidemiol. 2004;32(5):345–53.

18. Solar O, Irwin A. A conceptual framework for action on the social determinants of health. Social Determinants of Health Discussion Paper 2 (Policy and Practice). World Health Organization Geneva. Geneva, World Health Organization; 2010.

19. Elley WB, Irving JC. Revised socio-economic index for New Zealand. New Zeal J Educ Stud. 1976;11:25–36.

20. Schaefer JD, Caspi A, Belsky DW, Harrington H, Houts R, Israel S, et al. Early-Life Intelligence Predicts Midlife Biological Age. J Gerontol B Psychol Sci Soc Sci. 2016 Nov;71(6):968–77.

21. Calvin CM, Deary IJ, Fenton C, Roberts BA, Der G, Leckenby N, et al. Intelligence in youth and all-cause-mortality: Systematic review with meta-analysis. Int J Epidemiol. 2010/10/29. 2011 Jun;40(3):626–44.

22. Sörberg Wallin A, Allebeck P, Gustafsson JE, Hemmingsson T. Childhood IQ and mortality during 53 years’ follow-up of Swedish men and women. J Epidemiol Community Health [Internet]. 2018 Oct 1 [cited 2020 Jun 30];72(10):926–32. Available from: https://pubmed.ncbi.nlm.nih.gov/29925669/

23. Thomson WM, Broadbent JM, Caspi A, Poulton R, Moffitt TE. Childhood IQ predicts age-38 oral disease experience and service-use. Community Dent Oral Epidemiol. 2019 Jun 1;47(3):252–8.

24. Wechsler D. Manual for the Wechsler intelligence scale for children - Revised. San Antonio, TX: The Psychological Corporation; 1974.

25. Caspi A, Houts RM, Ambler A, Danese A, Elliott ML, Hariri A, et al. Longitudinal assessment of mental health disorders and comorbidities across 4 decades among participants in the Dunedin birth cohort study. JAMA Netw open. 2020;3(4):1–14.

26. Elliott ML, Caspi A, Houts RM, Ambler A, Broadbent JM, Hancox RJ, et al. Disparities in the pace of biological aging among midlife adults of the same chronological age have implications for future frailty risk and policy. Nat Aging. 2021 Mar 15;1(3):295–308.

27. Belsky DW, Caspi A, Houts R, Cohen HJ, Corcoran DL, Danese A, et al. Quantification of biological aging in young adults. Proc Natl Acad Sci U S A. 2015 Jul 28;112(30):E4104–10.

28. Centers for Disease Control and Prevention. Defining adult overweight and obesity. [Internet]. 2022. Available from: https://www.cdc.gov/obesity/basics/adult-defining.html

29. World Health Organization. Waist circumference and waist-hip ratio. Report of a WHO Expert Consultation. Geneva: World Health Organization; 2008.

30. National cholesterol education program (NCEP) expert panel on detection, evaluation, and treatment of high blood cholesterol in adults (Adult Treatment Panel III) final report. Vol. 106, Circulation. United States; 2002 Dec.

31. Sierra-Johnson J, Romero-Corral A, Lopez-Jimenez F, Gami AS, Sert Kuniyoshi FH, Wolk R, et al. Relation of Increased Leptin Concentrations to History of Myocardial Infarction and Stroke in the US Population. Am J Cardiol [Internet]. 2007 Jul 7 [cited 2022 Sep 19];100(2):234. Available from: /pmc/articles/PMC2000836/

32. Whelton PK, Carey RM, Mancia G, Kreutz R, Bundy JD, Williams B. Harmonization of the American College of Cardiology/American Heart Association and European Society of Cardiology/European Society of Hypertension Blood Pressure/Hypertension Guidelines: Comparisons, Reflections, and Recommendations. Circulation [Internet]. 2022 Sep 13 [cited 2022 Sep 16];146(11):868–77. Available from: https://www.ahajournals.org/doi/abs/10.1161/CIRCULATIONAHA.121.054602

33. Belsky DW, Caspi A, Israel S, Blumenthal JA, Poulton R, Moffitt TE. Cardiorespiratory fitness and cognitive function in midlife: neuroprotection or neuroselection? Ann Neurol [Internet]. 2015 Apr 1 [cited 2021 Jun 3];77(4):607–17. Available from: https://pubmed.ncbi.nlm.nih.gov/25601795/

34. Israel S, Moffitt TE, Belsky DW, Hancox RJ, Poulton R, Roberts B, et al. Translating personality psychology to help personalize preventive medicine for young adult patients. J Pers Soc Psychol. 2014 Mar;106(3):484–98.

35. Rabe KF, Hurd S, Anzueto A, Barnes PJ, Buist SA, Calverley P, et al. Global strategy for the diagnosis, management, and prevention of chronic obstructive pulmonary disease: GOLD executive summary. Am J Respir Crit Care Med. 2007 Sep 15;176(6):532–55.

36. Expert panel report: Guidelines (2013) for the management of overweight and obesity in adults. Vol. 22, Obesity. Blackwell Publishing Inc.; 2014.

37. Grundy SM, Stone NJ, Bailey AL, Beam C, Birtcher KK, Blumenthal RS, et al. 2018 AHA/ACC/AACVPR/AAPA/ABC/ACPM/ADA/AGS/APhA/ASPC/NLA/PCNA Guideline on the Management of Blood Cholesterol: A Report of the American College of Cardiology/American Heart Association Task Force on Clinical Practice Guidelines. J Am Coll Cardiol. 2019;73(24):e285–350.

38. Carnevale Schianca GP, Pedrazzoli R, Onolfo S, Colli E, Cornetti E, Bergamasco L, et al. ApoB/apoA-I ratio is better than LDL-C in detecting cardiovascular risk. Nutr Metab Cardiovasc Dis. 2011 Jun 1;21(6):406–11.

39. Matsushita K, van der Velde M, Astor BC, Woodward M, Levey AS, de Jong PE, et al. Association of estimated glomerular filtration rate and albuminuria with all-cause and cardiovascular mortality: a collaborative meta-analysis of general population cohorts. Lancet [Internet]. 2010 Jun 6 [cited 2022 Sep 19];375(9731):2073. Available from: /pmc/articles/PMC3993088/

40. Pearson TA, Mensah GA, Alexander RW, Anderson JL, Cannon RO, Criqui M, et al. Markers of inflammation and cardiovascular disease: Application to clinical and public health practice: A statement for healthcare professionals from the centers for disease control and prevention and the American Heart Association. Circulation [Internet]. 2003 Jan 28 [cited 2022 Sep 19];107(3):499–511. Available from: https://www.ahajournals.org/doi/abs/10.1161/01.cir.0000052939.59093.45

41. Dean L. Blood groups and red cell antigens [Internet]. Bethesda, Md: National Center for Biotechnology Information (US); 2005 [cited 2022 Sep 19]. Available from: https://www.ncbi.nlm.nih.gov/books/NBK2261/
